# A Unique Case of Prostate Carcinoma Presenting as Retroperitoneal Lymphadenopathy With Normal Levels of Prostate-Specific Antigen and a Prostate of Normal Size: A Case Report

**DOI:** 10.7759/cureus.52962

**Published:** 2024-01-25

**Authors:** Swapnil Patil, Mayank Mundada, Pradnya M Diggikar, Raju Hansini Reddy, Sree Vidya Yekkaluru

**Affiliations:** 1 Internal Medicine, Dr. D. Y. Patil Medical College, Hospital and Research Centre, Pune, IND

**Keywords:** conglomerated lymph node mass, prostate biopsy, normal prostate size, retroperitoneal lymphadenopathy, metastatic prostate carcinoma, prostate-specific antigen (psa)

## Abstract

Despite the significant advancements in prostate-specific antigen (PSA) screening and the diverse array of available treatments, prostate cancer (PCa) still significantly contributes to cancer-related illness. The most prevalent sites for metastases are bones, distant lymph nodes, and abdominal organs. Nevertheless, metastasis to the renal and retroperitoneal regions originating from prostate cancer constitutes an exceptionally uncommon clinical occurrence. Metastatic PCa commonly presents with elevated serum PSA levels, a hallmark of its diagnostic profile. However, there are instances where patients exhibit atypical metastatic patterns or maintain normal PSA levels. In the case under consideration, the patient exhibited a periureteral tumor with an indeterminate primary origin, subsequently confirmed to be metastatic prostate cancer.

This case underscores the importance of recognizing the varied and sometimes elusive presentations of metastatic PCa. Despite its rarity, the occurrence of renal and retroperitoneal metastasis emphasizes the need for vigilance and a comprehensive understanding of the diverse manifestations of advanced PCa for timely and accurate diagnosis, which is paramount in optimizing patient care and outcomes.

## Introduction

Prostate cancer (PCa) stands as the most common solid cancer affecting males globally. The prevailing method for PCa screening involves assessing elevated levels of prostate-specific antigen (PSA), currently considered the gold standard. Diagnosis is confirmed through a prostate needle biopsy [1]. However, false positives may occur because of benign prostate enlargement, prostatitis, or physiological changes [2]. PCa can sometimes occur in men with normal PSA levels [3]. Most patients with metastatic PCa have bone metastases and elevated PSA levels. PCa rarely metastasizes to the retroperitoneum. The presence of metastases primarily determines PCa prognosis. Detecting PCa metastasis has not been standardised, and many imaging modalities, including CT, bone scintigraphy, MRI, the newly developed prostate-specific membrane antigen (PSMA) and positron emission tomography/computed tomography (PET/CT) can be employed in the diagnosis and staging of PCa [1]. For cases characterized by localized disease, the primary treatment modalities involve surgery and radiotherapy. In instances of recurrent or metastatic disease, the predominant medical interventions include androgen-deprivation therapy (ADT), androgen signaling inhibition (ARSI), and chemotherapy. Nevertheless, a substantial proportion of individuals eventually develop castration resistance, a condition linked with an unfavorable prognosis [4]. The Gleason score is used for histopathological staging, which is combined with clinical staging for prognosis and treatment.

This case report outlines the clinical presentation of a 67-year-old male who presented to us with non-calculus mild hydroureteronephrosis on both sides and retroperitoneal lymphadenopathy, with normal PSA and a normal-sized prostate, misleading the diagnosis of retroperitoneal lymphoma; however, a subsequent lymph node biopsy confirmed the diagnosis of metastatic adenocarcinoma originating from the prostate.

## Case presentation

A 67-year-old male presented with bilateral pitting pedal edema, not associated with any redness or tenderness, for one month. The patient complained of an increased frequency of micturition for six months. No complaints of dribbling of urine, hesitancy, urgency, or altered urinary stream. The patient has had a known case of hypertension for 10 years and is on tablet telmisartan 40 mg once daily. No other past history was significant. On examination, the pulse rate was 70 bpm, and the blood pressure was 120/70 mm of hg. Systemic examination revealed no abnormalities.

Laboratory investigations (Table 1) revealed mildly deranged renal function tests and raised erythrocyte sedimentation rate.

**Table 1:** Laboratory investigations on the day of presentation. SGOT- Serum glutamic oxaloacetic transaminase SGPT- Serum glutamate pyruvate transaminase ESR- Erythrocyte sedimentation rate TSH- Thyroid stimulating hormone T3- Triiodothyronine T4- Thyroxine HbA1C- Hemoglobin A1c

Parameters [normal limit]	Report
Haemoglobin (13.2-16.6 gm/dl)	13.3 gm/dl
Total leucocyte count (4,000-10,000 /µL)	5100 /µL
Platelet count (1,50,000-4,10,000 /µL)	1,60,000 /µL
Serum urea (17–49 mg/dL)	42 mg/dL
Serum creatinine (0.6–1.35 mg/dL)	1.7 mg/dL
Serum bilirubin (0.2–1.2 mg/dL)	0.7 mg/dL
SGOT (8–48 IU/L)	33 IU/L
SGPT (7–55 IU/L)	23 IU/L
ESR (up to 20 mm/hr)	63 mm/hr
Random blood sugar level (up to 140 mg/dl)	123 mg/dl
Total protein (6.0-8.3 g/dl)	7.2 g/dl
Serum albumin (3.4-5.0 g/dl)	4.0 g/dl
Serum globulin (2.3-3.5 g/dl)	3.2 g/dl
Serum sodium (136-145 mmol/Lt)	140 mmol/Lt
Serum potassium (3.50-5.10 mmol/Lt)	3.6 mmol/Lt
Serum chloride (98-107 mmol/Lt)	101 mmol/Lt
TSH (0.35-4.94 uIU/L)	1.8 uIU/L
T3 (0.64-1.52 ng/ml)	0.7 ng/ml
T4 (4.87-11.72 ug/dl)	5.7 ug/dl
Urine routine microscopy	Within normal limit
HbA1C (<5.7%)	5.4%
Serum prostate specific antigen (<4.5ng/ml)	3.5ng/ml

Ultrasonography of the abdomen and pelvis revealed moderate hydronephrosis and mild hydroureter on both sides, with no other obvious abnormalities.

**Figure 1:**
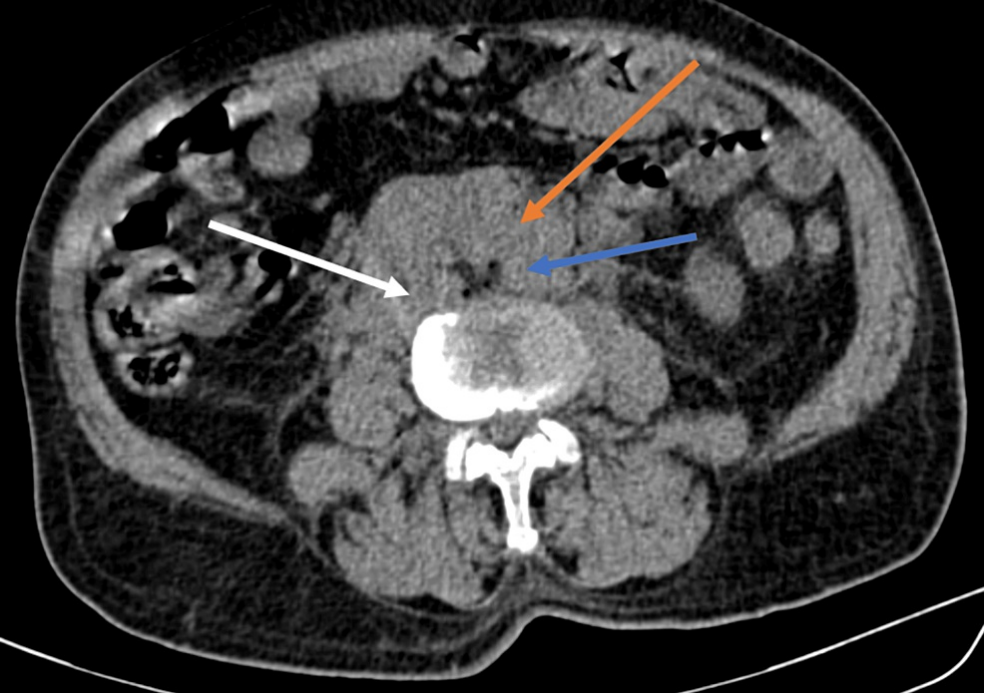
Transverse section of computed tomography of kidney, ureter, bladder showing a large multilobulated soft tissue density mass (Orange arrow) noted in retroperitoneum completely encasing aorta (Blue arrow) suggestive of a conglomerated lymph nodal mass. The mass is also causing variable external mass effect/encasement of mid-portion of both ureters leading to bilateral proximal moderate hydroureteronephrosis. White arrow indicates inferior vena cava.

**Figure 2:**
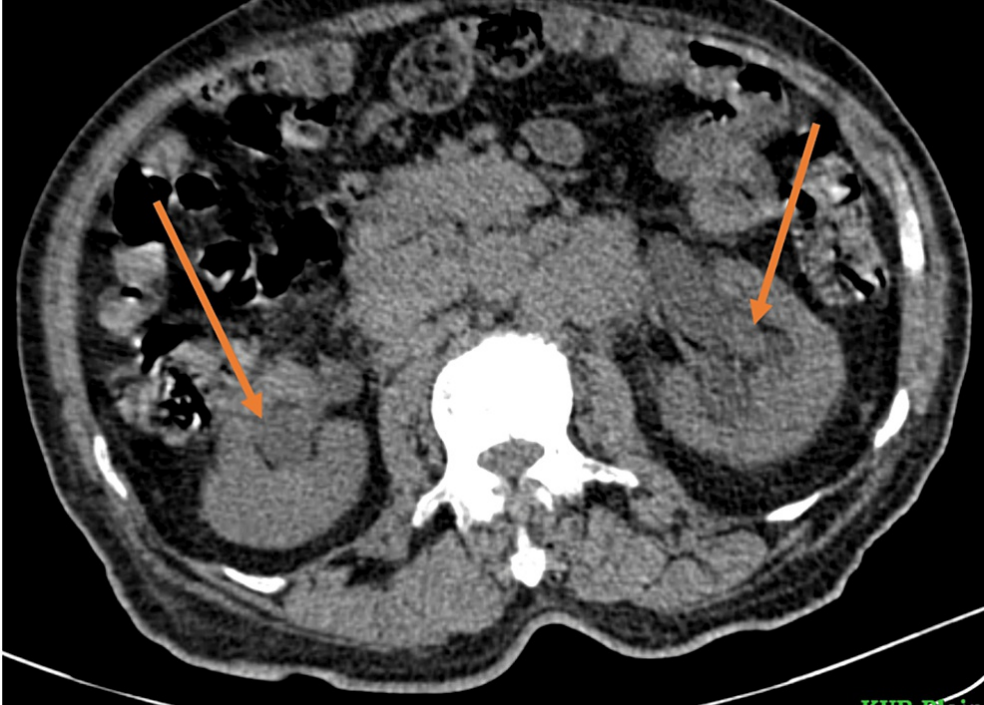
Transverse section of computed tomography of kidney, ureter, bladder indicating bilateral hydronephrosis (Orange arrows).

**Figure 3:**
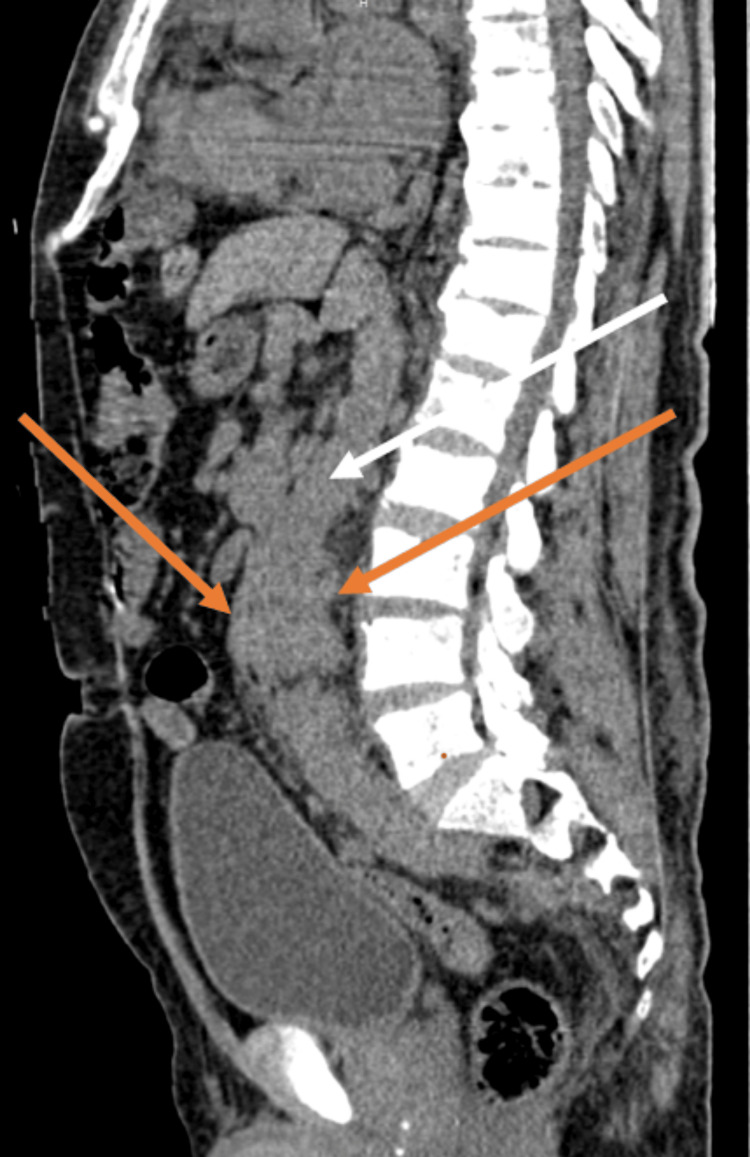
Sagittal section of computed tomography of kidney, ureters and bladder showing hypodense mass lesion circumferentially encasing the descending aorta (Orange arrows) and white arrow indicates the descending aorta.

**Figure 4:**
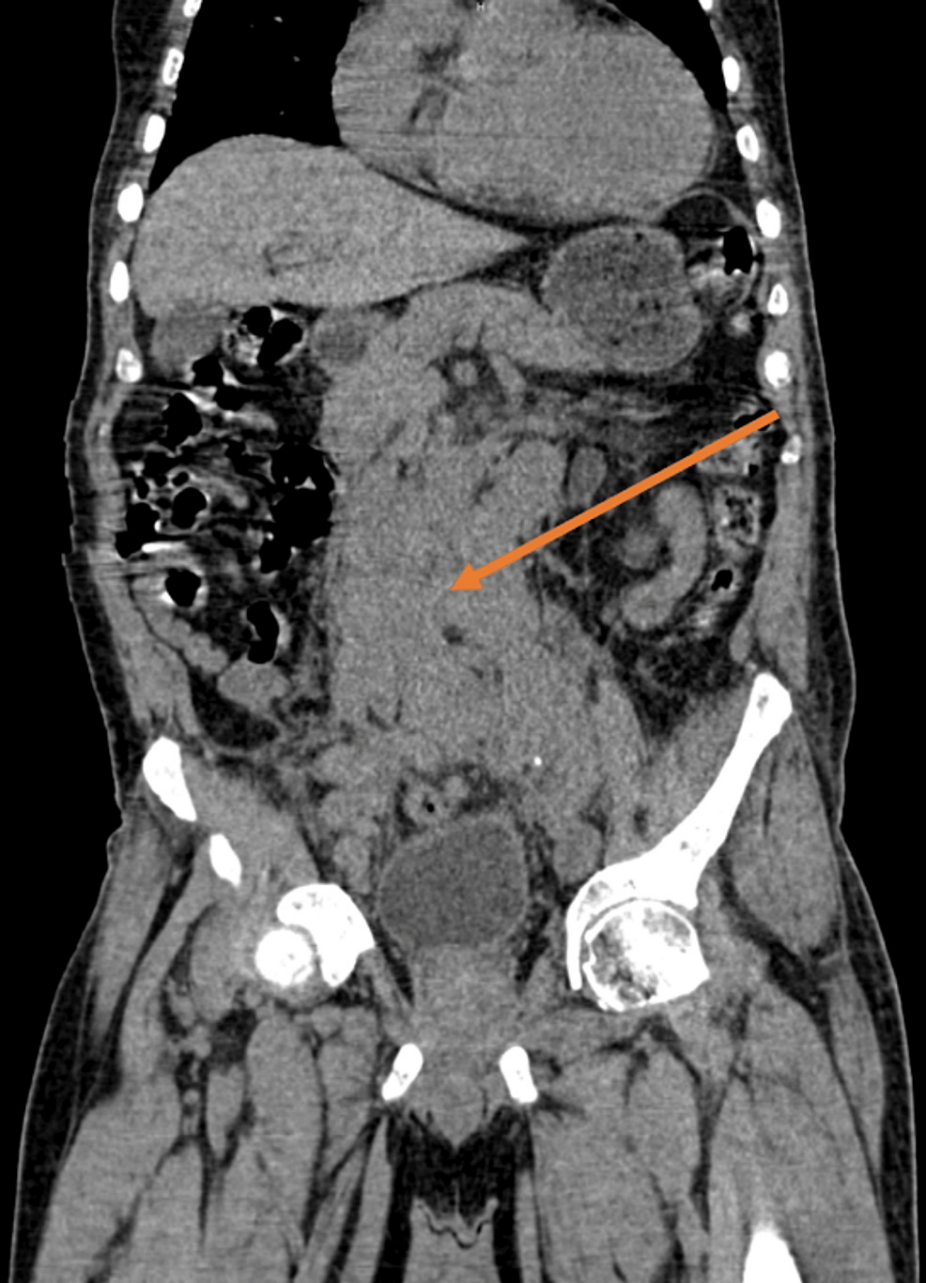
Coronal section of computed tomography of kidney, ureters and bladder indicating large hypodense mass (Orange arrow).

Computed tomography of the kidney, ureter, and bladder (Figures 1-4) revealed a large multilobulated soft tissue density mass measuring 5x9x2.1 cm (anterioposterior x transverse x craniocaudal) noted in the retroperitoneum completely encasing the aorta, bilateral renal veins, bifurcation of the aorta, and common iliac vessels suggestive of conglomerated lymph node mass causing mass effect on mid portions of both ureters leading to bilateral proximal moderate hydro-uretero-nephrosis. The prostate was normal in size.

**Figure 5:**
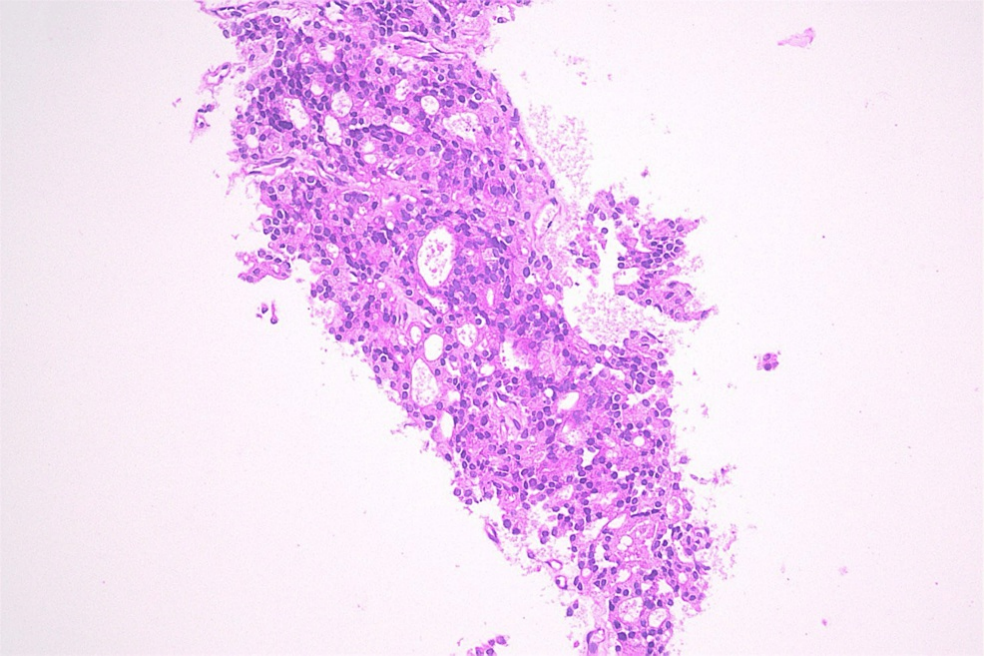
Histological staining using hematoxylin and eosin of the lymph node biopsy specimen illustrating prostate adenocarcinoma.

**Figure 6:**
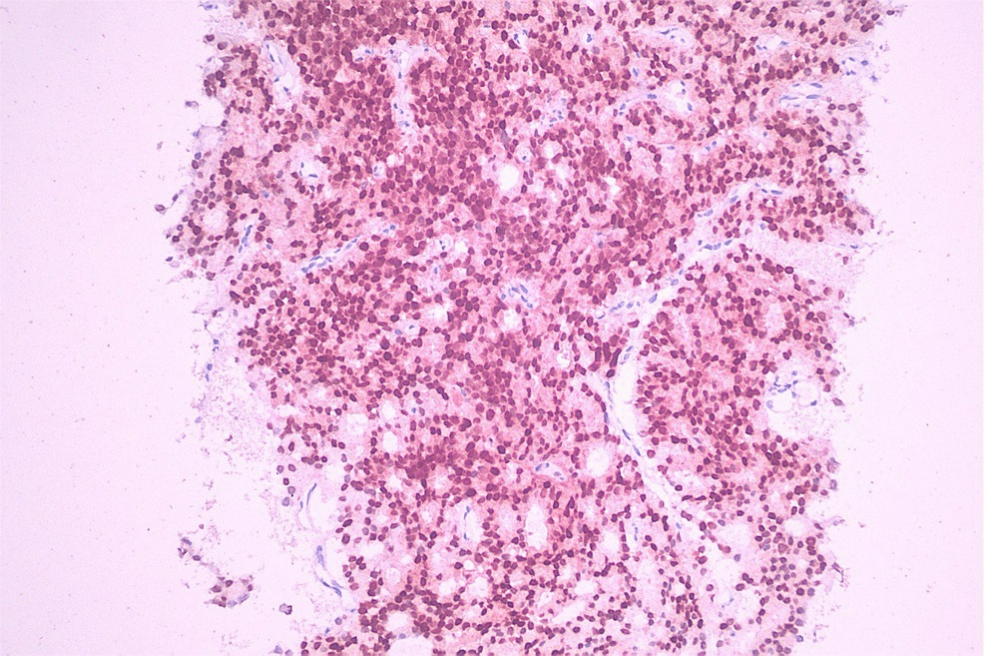
Immunohistochemistry analysis of the lymph node biopsy specimen demonstrating positive expression of NKX3.1 in prostate carcinoma.

Computed tomography-guided Tru-Cut biopsy of the lymph node mass was performed, which showed metastatic prostate adenocarcinoma on hematoxylin and eosin staining (Figure 5) with positive cytokeratin AE1/AE3, homeobox protein NKX3.1 (Figure 6), alpha-methyl acyl-CoA racemase and synaptophysin immunohistochemistry (Table 2).

**Table 2:** Immunohistochemistry of lymph node biopsy specimen. CK- Cytokeratin AE- Monoclonal antibody NKX3.1- Homeobox protein Nkx-3.1 AMACR- Alpha-methyl acyl-CoA racemase TTF1- Thyroid transcription factor-1 Pax8- Paired box gene 8 CdX2- Homeobox protein CDX-2

Test	Result
CK (AE1/AE3)	Diffusely positive
NKX3.1	Diffusely Positive
AMACR	Diffusely positive
Synaptophysin	Focally positive
CK7/CK20	Negative
TTF1/Pax8/CdX2	Negative

According to the findings outlined above, the patient received a diagnosis of metastatic adenocarcinoma of the prostate. However, the patient was referred to the oncology department for further management, but subsequently was lost to follow-up.

## Discussion

PCa ranks as the second most common malignancy among men in the Western world and stands as the second leading contributor to cancer-related mortality among men on a global scale [5]. While PCa typically follows a well-established pattern of spreading, often to regional lymph nodes and the skeleton, there is a subset of patients who may exhibit atypical metastases at the point of diagnosis. In research by Giorgio et al., an examination of 74,826 individuals diagnosed with metastatic PCa between 1998 and 2010 revealed that the predominant sites of metastasis included the bone (84%), distant lymph nodes (10.6%), liver (10.2%), and thorax (9.1%) [6]. According to research findings, 94.7% of individuals with advanced PCa demonstrated pelvic lymph node invasion in a study involving 19 participants. In the pelvic nodal sites affected, the obturator demonstrated the highest occurrence at 88.8%, succeeded by the external iliac (83.3%), common iliac (77%), hypogastric (44.4%), and presacral (33.3%) locations. Additionally, retroperitoneal lymph node involvement was reported by 77.8% of the participants (14 individuals) [7]. Early PCa is usually asymptomatic, and an elevated serum PSA level frequently serves as the initial indicator of malignancy. In the early detection of PCa, serum prostate-specific antigen has shown markedly superior effectiveness in comparison to digital rectal examination (DRE) and transrectal prostatic ultrasonography (TRUS) [8]. Previous studies have indicated a correlation between elevated PSA levels and the identification of metastatic PCa. A positive predictive value of 65% has been demonstrated for both skeletal involvement and metastatic disease when serum PSA concentrations exceed 20 ng/mL [8]. As reported in a study, the collective sensitivity, specificity, and positive predictive value for PSA were established at 72.1%, 93.2%, and 25.1%, respectively. The study further demonstrated that in cases where PSA levels fall within the normal range, there is approximately a 10% probability of overlooking cancer [9]. In research conducted by Thompson et al., the investigation focused on the prevalence of PCa in males with PSA levels below 4 ng/mL. The prevalence was roughly estimated to be 15%, revealing a disease risk of 10.1% in patients with PSA levels ranging from 0.6 to 1 ng/ml. This risk increased to 26.9% for individuals with PSA levels between 3.1 and 4 ng/ml [10]. Although the occurrence of normal PSA results in the presence of prostate cancer is deemed unusual, it has been reported in the literature that this occurs in approximately 20% of instances [11].

## Conclusions

The management of PCa poses a significant challenge for clinicians because of its atypical presentation. Patients have been reported to have uncommon manifestations, such as osteolytic bone metastases, a prominent abdominal lump, peritoneal metastasis accompanied by malignant ascites, and cervical lymphadenopathy. In addition, PCa can also manifest as pneumothorax or anejaculation. Of particular note is the rare occurrence of PCa presenting solely with retroperitoneal lymphadenopathy, despite maintaining normal serum PSA levels. This uncommon manifestation highlights the significance of contemplating PCa as a potential diagnosis, even when the PSA levels are normal. Recognizing this atypical manifestation of metastatic PCa is crucial in order to minimize diagnostic delays and enhance overall survival rates.

In summary, clinicians should uphold a heightened level of suspicion for PCa in the differential diagnosis when evaluating men with a retroperitoneal mass. A comprehensive understanding of the diverse presentations of PCa, including the rare instances described above, is essential for prompt and accurate diagnosis, ultimately contributing to improved patient outcomes.
